# Clinical review: Statins and trauma - a systematic review

**DOI:** 10.1186/cc12499

**Published:** 2013-05-29

**Authors:** Jan O Jansen, Janet M Lord, David R Thickett, Mark J Midwinter, Daniel F McAuley, Fang Gao

**Affiliations:** 1 Departments of Surgery and Intensive Care Medicine, Aberdeen Royal Infirmary; and University of Aberdeen, Aberdeen AB25 2ZN, UK; 2 MRCARUK Centre for Musculoskeletal Ageing Research, Centre for Translational Inflammation Research, Queen Elizabeth Hospital, University of Birmingham, B15 2WB, UK; 3 Perioperative, Critical Care and Trauma Trials Group, Centre for Translational Inflammation Research, Queen Elizabeth Hospital, University of Birmingham, B15 2WB, UK; 4 NIHR Surgical Reconstruction and Microbiology Research Centre, University of Birmingham, University Hospital Birmingham; and Academic Department of Surgery and Trauma, Royal Centre for Defence Medicine, Birmingham B15 2SQ, UK; 5 Centre for Infection and Immunity, Queen's University of Belfast; and Intensive Care Unit, Royal Victoria Hospital, Belfast, BT9 7AE, UK

## Abstract

Statins, in addition to their lipid-lowering properties, have anti-inflammatory actions. The aim of this review is to evaluate the effect of pre-injury statin use, and statin treatment following injury. MEDLINE, EMBASE, and CENTRAL databases were searched to January 2012 for randomised and observational studies of statins in trauma patients in general, and in patients who have suffered traumatic brain injury, burns, and fractures. Of 985 identified citations, 7 (4 observational studies and 3 randomised controlled trials (RCTs)) met the inclusion criteria. Two studies (both observational) were concerned with trauma patients in general, two with patients who had suffered traumatic brain injury (one observational, one RCT), two with burns patients (one observational, one RCT), and one with fracture healing (RCT). Two of the RCTs relied on surrogate outcome measures. The observational studies were deemed to be at high risk of confounding, and the RCTs at high risk of bias. Three of the observational studies suggested improvements in a number of clinical outcomes in patients taking statins prior to injury (mortality, infection, and septic shock in burns patients; mortality in trauma patients in general; mortality in brain injured patients) whereas one, also of trauma patients in general, showed no difference in mortality or infection, and an increased risk of multi-organ failure. Two of three RCTs on statin treatment in burns patients and brain injured patients showed improvements in E-selectin levels and cognitive function. The third, of patients with radial fractures, showed no acceleration in fracture union. In conclusion, there is some evidence that pre-injury statin use and post-injury statin treatment may have a beneficial effect in patients who have suffered general trauma, traumatic brain injury, and burns. However, these studies are at high risk of confounding and bias, and should be regarded as 'hypothesisgenerating'. A well-designed RCT is required to determine the therapeutic efficacy in improving outcomes in this patient population.

## Introduction

Trauma is the leading cause of death in under 40 year olds [1], Many of these patients die from exsanguination or devastating neurological injury, but those who survive the initial insult often do not succumb to their injuries *per se*, but die as a result of overwhelming inflammatory dysregulation, leading to organ dysfunction, nosocomial infection, and ultimately, multiorgan failure [2-8], Following major injury there is a dynamic balance between promoters and inhibitors of both alarm and subsequent stress responses in the inflammatory system that may be adaptive (beneficial) or maladaptive (harmful), There has been no evolutionary selection pressure to create an adaptive response to major injury, so although body systems are exquisitely balanced to respond to minor to moderate injury, the response to major injury is not necessarily beneficial. In major trauma this sequence is linked to the development of a systemic inflammatory response syndrome, which is the result of activation of the innate immune system [4,9,10],

The presence of such a response, on admission, is associated with higher mortality, an increased incidence of multiple organ dysfunction syndrome, and higher rates of sepsis [11-13], Modulation of the immune system after major traumatic injury is therefore conceptually attractive, and has the potential to reduce morbidity and mortality from major trauma, but is also a complex and controversial proposition.

3-Hydroxy-3-methylglutaryl-coenzyme A (HMG-CoA) reductase mediates the reduction of HMG-CoA, one of the precursors of cholesterol synthesis, Inhibitors of this enzyme, commonly known as 'statins', are structural mimics of HMG-CoA and compete for binding to the enzyme and inhibit the production of mevalonate and subsequently cholesterol [14]. The reduced production of cholesterol in the liver results in the upregulation of low density lipoprotein receptors on hepatocytes with increased capture of circulating cholesterol [15].

Although the beneficial effects of statins have been primarily ascribed to their lipid-lowering properties [14], more recently their anti-inflammatory actions have been recognised and are now thought to contribute significantly to their disease modifying effects [16]. That statin's effects may include actions beyond cholesterol reduction is suggested by the observation that a range of statin preparations appear to show similar efficacy with regard to cholesterol reduction in cardiovascular morbidity or mortality, despite differing abilities to reduce serum cholesterol [17]. In addition, statins have been shown to reduce morbidity in patients who did not have high serum cholesterol or cardiovascular disease, but did have evidence of systemic inflammation [18]. The anti-artherogenic actions of statins include improved endothelial function [19], with reduced thrombus formation [20] and improved atherotic plaque stability [21], as well as the modulation of inflammatory responses [16,22,23]. Interestingly, conditions where statins have been found to have a positive effect on disease progression or mortality are primarily dependent on leucocyte accumulation [24]. Statins may thus promote the timely resolution of the inflammatory response, preventing persistence of inflammation and resultant pathology.

There is now growing agreement that many of the beneficial effects of statins are dependent on HMG-CoA reductase inhibition. Protein prenylation is required for the normal function of small GTPases such as Rho and this is prevented by HMG-CoA reductase inhibition. In this way statins have wide ranging effects on cellular processes such as cell migration, reactive oxygen species generation and secretion of proinflammatory cytokines that contribute to the modulation of the inflammatory response [25], and there is evidence from human and animal studies that these drugs may have beneficial effects in a number of conditions characterised by excessive inflammation [26,27]. There are also an increasing number of reports of favourable effects in patients who have suffered injury, thought to also be mediated by effects on inflammation and the immune system.

The aim of this systematic review is to examine the evidence for beneficial effects of pre-injury statin use and post-injury statin treatment in trauma patients. It will consider trauma in general, selected single-system injuries (fractures and traumatic brain injury), as well as burns patients. It will not consider the broader issues of the effect of statin treatment in critically ill patients, although it is acknowledged that a beneficial effect in trauma patients, if present, may be related to improved outcomes from sepsis or acute lung injury.

## Materials and methods

### Research question

This review aims to answer the question: 'Does pre-injury statin use or post-injury statin treatment improve mortality, or functional outcomes, or reduce complications in patients who have suffered trauma in general, traumatic brain injury, burns, or fractures?'

### Data sources and search strategy

We searched MEDLINE, EMBASE, and the Cochrane Central Database of Controlled Trials for reports published up to January 2012 to identify studies for inclusion in this review. The search was limited to studies involving humans. No language restriction was imposed. The search terms included ['hydroxymethylglutaryl-CoA reductase inhibitors', 'statin', 'atorvastatin', 'simvastatin', 'pravastatin', 'fluvastatin', or 'lovastatin']; and ['fracture healing' or 'fracture'], or ['brain injuries' or 'traumatic' and 'brain' and 'injury'], or ['burn'], or ['wounds and injuries' or 'injury']. Both medical subject heading (MeSH) and free text searches were performed. We also searched the proceedings of trial databases, and the reference lists of identified trials and major reviews.

### Study selection

One reviewer (JOJ) examined the titles and abstracts of the electronic search results to identify articles that were obviously irrelevant. Two reviewers (JOJ, and DRT or MJM) then independently examined the full text articles of the remaining studies to determine eligibility. We included both randomised and observational studies, but excluded case series without control groups.

### Study appraisal

Given the poor agreement on the use of summary scores and checklists, the quality of the included studies was assessed individually, by methodological domain. For randomised studies, we considered sequence generation, allocation concealment, blinding of participants, personnel, and outcomes assessment, attrition, and selective reporting. For non-randomised studies, we considered the type of comparison, identification of participants, participant allocation, and risk of confounding.

## Results

### Trauma in general

#### Study characteristics

Our search identified 526 articles, 524 of which were excluded following review of the titles and abstracts. Two articles, both non-randomised studies, were eligible for full-text review, and are summarised in Table 1.

**Table 1:** Observational studies

Study	Design	Participants	Exposure	Comparisons	Outcome	Results	Subgroup analyses	Remarks
Fogerty *et al*. (2010) [32]	Retrospective cohort study	223 patients, aged ≥55 years, with thermal burns, admitted to a regional burns centre	Pre-injury statin use (n = 70), duration not specified, continued after hospitalisation in 77%	No pre-injury statin use (n = 153)	In-hospital mortality Infection Septic shock	OR 0.17 (95% CI 0.05-0.57) OR 0.90 (95% CI 0.48-1.7) OR 0.50 (95% CI 0.20-1.30)	No change in odds ratio when stratified by cardiovascular comorbidities	Statin therapy continued after hospitalisation in 77% Multivariate regression analysis determined odds ratios of death and sepsis by statin use, adjusting for cardiovascular comorbidities
Efron *et al*. (2008) [28]	Retrospective cohort study	1,224 patients, aged 65-84 years with moderate-severe traumatic injury (AIS ≥3), survival >24 h, participating in NSCOT study	Pre-injury statin use (21.1%), duration not specified, continuation after admission not known	No pre-injury statin use (78.9%)	In-hospital mortality	OR 0.33 (95% CI 0.12-0.92)	Subgroup with cardiovascular comorbidity (n = 414): OR 1.41 (95% CI 0.72-2.72) Subgroup without cardiovascular comorbidity (n = 775): OR 0.30 (95% CI 0.10-0.91)	Multivariate logistic regression analysis NSCOT study captured pre-injury medication by class only. No data on compliance, duration, dose, or whether continued after admission to hospital NSCOT study used very complex statistical sampling model
Neal *et al*. (2009) [29]	Retrospective cohort study	295 patients, aged 55-90 years, blunt mechanism of injury, hypotension (systolic blood pressure <90 mmHg) or biochemical evidence of hypoperfusion (base deficit >5 meq/L) on admission, blood transfusion requirement, at least one AIS ≥2 other than head, survival >24 h, participating in Host Response to Injury Large Scale Collaborative Program	Pre-injury statin use (n = 71), as verified by patient or relative	No pre-injury statin use (n = 224)	In-hospital mortality Nosocomial infection (microbiologically confirmed pneumonia, catheter-related bloodstream infection or urinary tract infection) Multi-organ failure (defined as Marshall score >5)	HR 1.98 (95% CI 0.9-4.0) HR 0.78 (95% CI 0.5-1.4) HR 1.81 (95% CI 1.1-2.9)		Propensity score adjusted regression analysis to control for differences in baseline characteristics No data on whether statin therapy was continued after hospital admission
Schneider *et al*. (2011) [30]	Retrospective cohort study	523 patients, aged 65 years and older, with head AIS ≥3, survival >24 h, participating in NSCOT study	Pre-injury statin use (22.3%), duration not specified, continuation after admission not known	No pre-injury statin use (77.7%)	In-hospital mortality Extended Glasgow Outcome Scale insurvivors at 3 and 12 months after injury	RR 0.24 (95% Cl 0.08-0.69) RR at 3 months 0.77 (95% Cl 0.42-1.41) RR at 12 months 1.13 (95% Cl 1.01-1.26)	Mortality subgroup with cardiovascular comorbidity: RR 0.87 (95% Cl 0.50-1.50) Subgroup without cardiovascular comorbidity: RR 0.17 (95% Cl 0.05-0.63)	Multivariate logistic regression analysis. NSCOT study captured pre-injury medication by class only. No data on compliance, duration, dose, or whether continued after admission to hospital. NSCOT study used very complex statistical sampling model

AIS, Abbreviated Injury Scale; CI, confidence interval; HR, hazard ratio; NSCOT, National Study on the Costs and Outcomes of Trauma; OR, odds ratio.

Efron and colleagues [28] conducted a retrospective cohort study based on data from the National Study on the Costs and Outcomes of Trauma (NSCOT) of 18 trauma centres and 51 non-trauma centre hospitals using a complex statistical sampling model. The authors used a sample weighting based on the conditional probability of being selected. The total weighted number of patients taking statins prior to injury was 529.2 (21.9%), compared with 1,887 (78.1%) who were not prescribed HMG-CoA reductase inhibitors. Pre-injury medication use was documented as part of routine care, and abstracted using a mapping program, to ten classes of medication, of which statins formed one. A major limitation of this study is lack of information on type, dose, duration of statin use and compliance. Furthermore, it was not clear whether pre-injury statin use was continued following hospital admission. Efron and colleagues [28] failed to include all adult patients in their analysis, because statin use in patients under 65 was limited only to 1.5% of the population, precluding a meaningful analysis. The analysis was therefore limited to subjects aged 65 to 84 years of age. A total of 1,224 such patients were identified for multivariate logistic regression analysis and subgroup analysis for presence/absence of cardiovascular comorbidities [28]. The risk of residual confounding is high.

The second retrospective cohort study, by Neal and colleagues [29], analysed the data of 295 patients with blunt trauma from the Host Response to Injury Large Scale Collaborative Program. These patients were older (aged 55 to 90 years) with severe blunt trauma, demonstrated by hypotension or hypoperfusion on admission. Casualties with isolated traumatic brain injuries and cervical cord injuries were excluded. Pre-injury medication use was recorded prospectively. Again, this study was also unable to ascertain whether pre-injury statin treatment was continued after admission. A propensity score predicting statin use was created using logistic regression to adjust for baseline differences in the cohorts. Cox proportional hazard regression was then used to evaluate the effects of pre-injury statin use on mortality, and the development of multiple organ failure and nosocomial infection [29]. Again, the risk of residual confounding is high.

#### Outcomes

Efron and colleagues [28] demonstrated that pre-injury statin use was associated with reduced odds of in-hospital mortality (odds ratio (OR) 0.33, 95% confidence interval (CI) 0.12 to 0.92). When stratified by the absence and presence of cardiovascular comorbidities, multivariable adjusted odds for statin use were 0.30 (95% CI 0.10 to 0.91) and 1.4 (95% CI 0.72 to 2.72), respectively [28]. In contrast, Neal and colleagues [29] failed to demonstrate an effect of pre-injury statin use on in-hospital mortality (hazard ratio (HR) 1.98, 95% CI 0.9 to 4.0) or on nosocomial infection (HR 0.78, 95% CI 0.5 to1.4). Furthermore, Neal and colleagues' study showed that pre-injury statin use was associated with an increased risk of multiple organ failure (HR 1.81, 95% CI 1.1 to 2.9) [29].

### Traumatic brain injury

#### Study characteristics

Our search identified 65 citations relating to traumatic brain injury. Examination of the abstracts categorised 63 as either irrelevant, or as case reports, reviews or editorials. These were excluded from further examination. Two clinical studies, one randomised controlled trial and one retrospective cohort study, underwent full text review [30,31] (Tables 1 and 2).

**Table 2:** Randomised studies

Study	Design	Participants	Intervention	Comparisons	Outcome	Results	Subgroup analyses	Remarks
Akcay *et al*. (2005) [33]	Clinical trial	20 patients with severe burns, treated in a single centre	Atorvastatin, 20 mg once daily, orally, for 14 days (n = 10)	Placebo (n = 10)	Plasma E-selectin	23.69 ng/ml (intervention group), 18.08 ng/ ml (control group) on enrolment; 10.86 ng/ ml and 21.69 ng/ml, respectively, after 14 days (*P *< 0.05)		
Tapia-Perez *et al*. (2008) [31]	Double blind randomised controlled trial, designated as 'pilot'	21 patients aged between 16 and 50 years with traumatic brain injury, GCS 9-13 and intracranial lesion on CT scan	Rosuvastatin, 20 mg once daily, for 10 days (n = 8)	Placebo (n = 13)	Reduction in amnesia time (measured using Galveston Orientation and Amnesia Test)	HR 53.76 (95% CI 1.58-1,824.64)		Despite randomisation, due to small numbers, the groups were not homogenous with regard to neurological parameters, with possibly more severe injury in treatment group
Patil *et al*. (2009) [46]	Double blind, placebo-controlled, randomised controlled trial	Patients with undisplaced, extra-articular distal radial fractures	Simvastatin, 20 mg, once daily (n = 31)	Placebo (n = 31)	Mean time to fracture union Mean percentage trabecular healing at 12 weeks post-injury	Simvastatin group, 71.7 days; control group, 71.3 days (*P *= 0.6481) Simvastatin group, 85%; control group, 87% (*P *= 0.431)		Low dose simvastatin used. No measures of simvastatin levels reported

AIS, Abbreviated Injury Scale; CI, confidence interval; GCS, Glasgow Coma Scale; HR, hazard ratio; NSCOT, National Study on the Costs and Outcomes of Trauma; RR, relative risk.

The study by Schneider and colleagues [30] was a retrospective cohort study, based on the collected data from the NSCOT. The selection criteria included an abbreviated injury scale of greater than 3 for the head region, and age greater than 65 years, as statin use in patients below this age was rare. Patients presenting with fixed dilated pupils or who died within 24 hours were excluded, as it was felt unlikely that statins would improve survival in this group. The data on pre-morbid medication use were incomplete. A total of 523 individuals met the inclusion criteria, 117 of whom were pre-injury statin users. After weighting (to allow for the fact that NSCOT included all patients who died in-hospital, but only a proportion of those who were discharged alive), 965 individuals were represented, 242 of whom were statin users. Outcomes include survival to discharge from hospital, and functional outcome at 3 and 12 months after injury, evaluated using the Extended Glasgow Outcome Scale. Multivariable modified Poisson regression analysis was performed to examine the relationship between pre-injury statin use and outcomes, accounting for age, new injury severity score, Glasgow Coma Scale, infection, shock, cardiovascular comorbidity (defined as a history of myocardial infarction, cerebrovascular or peripheral vascular disease, atrial or ventricular tachyarrhythmias), congestive cardiac failure, gender, beta-blocker use, renal disease, hypertension, mid-line shift on head CT scan, tobacco use, chronic obstructive pulmonary disease, diabetes mellitus and whether the patient was admitted to a trauma centre or not. A significant interaction between statin use and cardiovascular comorbidity was observed and a term defined by this interaction was included in the regression models. The interaction between statin use and cardiovascular comorbidity was further examined in analyses that stratified statin users by the presence or absence of underlying cardiovascular disease. Nevertheless, the risk of residual confounding is high, particularly given the unknown validity of the weighting algorithm.

The study by Tapia-Perez and colleagues [31], on statin treatment rather than pre-injury statin use, was a double blinded randomised controlled trial of rosuvastatin (20 mg, enterally, once daily) in patients who had suffered traumatic brain injury. The authors categorised the study as a 'pilot', to evaluate a possible positive effect of statin treatment on amnesia and disorientation after head injury. Inclusion criteria were patients aged between 16 and 50 years, with a Glasgow Coma Scale between 9 and 13, and an intracranial lesion demonstrated on CT scan. There were extensive exclusion criteria, including death within 72 hours, or due to causes other than brain injury, head injury within 4 weeks or disability due to neurological or psychiatric disease, multisystem trauma, grade III-IV hypovolaemic shock, surgical management of intracranial lesion, isolated brain stem injury, prior treatment elsewhere, pregnancy, and concomitant medication use. Forty-three patients were assessed for eligibility, of whom 22 were randomised. The primary outcome was the probability of a positive Galveston Orientation and Amnesia Test score to day 120. Secondary outcomes included cytokine levels at day three, and Disability Rating Scale assessment at three months. Participants and investigators were blinded. Follow-up, to three months, was complete. Analysis was by a Cox regression model. One patient died secondary to complicated abdominal surgery and was subsequently excluded from the analysis. Eight patients received rosuvastatin and 13 received placebo over a period of 10 days post-injury. The groups were incompletely matched for neurological criteria as more patients in the rosuvastatin group had absent pupillary reflexes compared to the placebo group, possibly suggesting more severe injury despite Glasgow Coma Scale scores being similar between the groups. Overall, this trial is thus also deemed at high risk of bias.

#### Outcomes

Schneider and colleagues [30] found that statin users were less likely to die before discharge than non-users(9.1% versus 15.4%, respectively). Multivariate regression demonstrated that, overall, statin users at time of injury had a 76% lower risk of dying before discharge(relative risk (RR) 0.24, 95% CI 0.08 to 0.69). Patient gender and concomitant beta-blocker use were not associated with mortality. This protective effect was greater in those without pre-injury cardiovascular morbidity; thus, statin use was associated with an 83% decrease in the risk of dying (RR 0.17, 95% CI 0.05 to 0.63). In contrast, there was no demonstrated protective effect in statin users with pre-injury cardiovascular morbidity (RR 0.87, 95% CI 0.05 to 1.50). In terms of functional recovery, as determined by extended Glasgow Outcome Scale, statin use was shown to be associated with benefit at 12 months post-injury (RR 1.13, 95% CI 1.01 to 1.26), although no benefit was demonstrated at three months post-injury (RR 0.77,95% CI 0.42 to 1.41), regardless of patients' pre-injury cardiovascular conditions.

Tapia-Perez and colleagues [31] demonstrated that rosuvastatin 20 mg per day (n = 8) improved Galveston Orientation and Amnesia Test score (HR 53.76, 95% CI 1.58 to 1,824.64) compared with placebo (n = 13). The effect was strongest for left-sided lesions, which may be related to memory and facial recognition being associated with right-sided cerebral structures. There was no difference in the Disability Rating Scale at three months between the two groups. This study indicates that statin given to victims after severe head injury may reduce the risk of developing amnesia.

### Burns

#### Study characteristics

Our search identified three articles, one of which was excluded following abstract review, leaving two full-text articles: one is an observational study, and the other a randomised controlled trial (Tables 1 and 2). We also identified one further, completed, randomised controlled trial, which has, however, not been published.

Fogerty and colleagues [32] conducted a retrospective cohort study of 223 consecutive burns patients, aged 55 years and over, admitted to a single regional burn centre, over a period of three years. Continuation of statin use following hospitalization was evaluated, and found to be 77%. Multivariate regression analysis was used to determine odds ratios of in-hospital mortality and septic shock, by pre-injury statin use, adjusting for cardiovascular comorbidities[32]. The study was susceptible to residual confounding.

Akcay and colleagues [33] conducted a prospective randomised controlled trial of atorvastatin treatment (20 mg, enterally, once daily, for 14 days) in 20 patients with burns who had not previously taken statins. The primary endpoint was plasma E-selectin, which the authors used as a biomarker of burn injury severity [33]. Patients were followed up to 14 days. The study was small, with no information on randomisation or allocation concealment, resulting in difficulties in assessing the quality of its methodology. This study is therefore also at high risk of bias.

#### Outcomes

Fogerty and colleagues [32] demonstrated decreased odds of in-hospital death in patients taking statins pre-injury (OR 0.17, 95% CI 0.05 to 0.57). This survival benefit remained unchanged when stratified by cardiovascular comorbidities. However, the authors failed to demonstrate a beneficial effect of pre-injury statin use on infection (OR 0.90, 95% CI 0.48 to 1.7) or septic shock (OR 0.50, 95% CI 0.20 to 1.30) [32]. Akcay and colleagues [33] demonstrated that, following 14 days' treatment, plasma E-selectin levels were significantly lower in the atorvastatin group compared with the placebo group (10.86 ng/ml versus 21.69 ng/ml, *P *< 0.05).

### Fracture healing

#### Study characteristics

Systematic searching for clinical studies identified 391 publications, of which 371 were excluded as irrelevant following review of the abstracts. Twenty articles underwent full text review. Four were excluded on methodological grounds. Of the 16 studies, 15 were concerned with fracture prevention, examining the effect of prior statin treatment on the risk of fractures in general, or certain types of fractures (in particular, hip fractures). All of these studies related to individuals at increased risk, such as older women with osteoporosis, or patients on haemodialysis. Nine retrospective studies demonstrated a benefit to statin use [34-42]. Two secondary analyses, of the LIPID and TIMI-22 trials, however, did not show a reduction in the risk of fracture in patients who had been randomised to statin treatment [43,44]. A dose-finding trial also failed to demonstrate a difference in bone mineral density in patients taking different doses of atorvastatin [45]. It is difficult to know whether the findings of these preventative studies can be extrapolated to the acute treatment of patients with injuries, and will therefore not be considered further in this review.

There is a single, randomised, double-blind, parallel, controlled trial reporting the effect of treatment with 20 mg simvastatin daily for 12 weeks on the time to distal radial fracture healing using dual-energy X-ray absorptiometry assessment of bone mineral density at 2 and 12 weeks post-injury (Table 2) [46]. There were extensive exclusion criteria. Sixty-two eligible patients were recruited (31 in each arm), although 18 were lost to follow-up and subsequently excluded, as only a per-protocol analysis was performed. This trial has a high risk of bias.

#### Outcomes

Simastatin 20 mg given post-fracture for 12 weeks did not improve fracture healing as the mean time to fracture union (*P *= 0.648) and the mean percentage trabecular healing at 12 weeks (*P *= 0.431) were the same in both groups [46]. The low dose of simvastatin used in this study may have contributed to the lack of effect, given that other studies of the non-lipid-lowering properties of statins have utilised higher doses [26,27].

## Discussion

The diagnosis 'trauma' encompasses a wide variety of direct injuries, associated complications, such as infection, and the consequences of treatment, such as lung injury caused by ventilation or blood transfusion. Patients who have suffered injury therefore constitute a highly heterogeneous group. Nevertheless, there are underlying mechanistic similarities that may be amenable to therapeutic intervention. Trauma is characterised by inflammation, both local and systemic, and the degree of inflammation is proportional to the magnitude of the combined insult of injury and treatment. A recent review of the epidemiology of civilian trauma deaths has shown that central nervous system injury continues to be the predominant cause of death after trauma (21.6 to 71.5%), followed by exsanguination (12.5 to 26.6%), sepsis (3.1 to17%) and multi-organ failure (1.6 to 9%) [47]. Over the past decade, a decrease (15 to 25%) in the proportion of haemorrhage-related deaths has been observed [47], in contrast to deaths due to traumatic brain injury, sepsis, and multi-organ failure, which have not decreased. These conditions are strongly related to excessive and injurious inflammation. Treatments to control or modulate the inflammatory response could help to mitigate against complications associated with these conditions. HMG-CoA reductase inhibitors are potentially attractive agents because they are generally safe and well tolerated drugs. As a result, treatment with statins is being investigated in a number of infective and inflammatory conditions, particularly in the critical care setting [48,49]. It is therefore not surprising that the possible effect of these drugs in trauma patients has also been considered.

This review has considered four separate groups of trauma patients, in whom pre-injury statin use, or post-injury statin treatment, has been evaluated. In total, there are only seven studies, four of which are observational. Three of these studies suggest improvements in a variety of clinical outcomes (mortality, infection, septic shock in burns patients; mortality in trauma patients in general; mortality in brain injured patients) in patients taking statins prior to injury, whereas one showed no difference in mortality or infection, and an increased risk of multi-organ failure, in general trauma patients. The design of these studies makes them susceptible to confounding, and in particular, the 'healthy user effect'. Further limitations were the age range and comorbidity of the participants, which was determined by the primary indication for commencing statin treatment. As a result, none of the observational studies included patients below the age of 55, and few below the age of 65.

The divergent results reported by the two non-randomised studies on trauma patients in general [28,29] are particularly intriguing. The differences may be explained by the inclusion criteria, suggesting that statins may exert different effects in specific groups of patients, possibly related to the type and magnitude of the inflammatory response present.

Several studies have described a 'rebound' effect from 'statin-withdrawal' after prolonged use, resulting in increased mortality after acute coronary syndrome [50], increased myonecrosis after vascular surgery [51], and an increased risk of perioperative cardiac events after dis-continuation [52]. Neither of the studies of statin use in trauma patients in general [28,29] were able to ascertain whether pre-injury statin use was continued after admission to hospital. This important confounder is therefore unaccounted for.

Two of three trials, of statin treatment in burns patients and brain injured patients, showed improvements in outcome, but relied on surrogate measures. The third study, of patients with fractures, demonstrated no acceleration in fracture union. All three trials had limitations, and are considered to be at high risk of bias. Again, there is insufficient evidence to support a specific type of statin or dose.

None of the studies included in this review reported adverse effects. Statin treatment for hypercholesterolaemia, in the outpatient setting, has been shown to be associated with few complications. However, statins can cause rhabdomyolysis, and it is not known whether trauma patients, or those with hypoperfusion, are more prone to this problem [53]. Statins are also known to have effects on the coagulation and fibrinolytic systems, which may underlie some of their protective effects in cardiovascular disease [54]. Consideration must therefore be given to the possibility of statins exacerbating acute traumatic coagulopathy. The effects of statins on haemostasis include diminished tissue factor activity, thrombin generation, and thrombin activity, reduced fi brinogen levels, and alterations in tissue plasminogen activator and plasminogen activitor inhibitor (PAI-1) activity [55,56]. Statins have also been shown to reduce platelet activity [57-59]. However, statins have not been reported to be associated with clinically significant coagulopathy in patients with trauma.

## Conclusion

There is some evidence from observational studies and small, randomised, prospective trials that statin treatment post-injury, or prior statin treatment, may have a beneficial effect in patients who have suffered general trauma, traumatic brain injury, or burns. However, these studies are at high risk of confounding and bias, and should be regarded as 'hypothesis-generating'. Animal studies may help to provide further proof of concept, but have intrinsic limitations. Ultimately, only a well-designed clinical trial will be able to answer the question of whether trauma patients may benefit from adjunctive HMG-CoA reductase inhibition. Designing such a trial will be challenging, and initial work should perhaps focus on statin-naïve patients who are likely to develop a pronounced inflammatory response. The difficulties in defining such a group are acknowledged. The possible role of statins in other conditions - in particular, severe sepsis and acute respiratory distress syndrome - are the subject of several ongoing, well-designed, large-scale, randomised controlled trials. The results of these studies are eagerly awaited, as these conditions also affect many severely injured patients.

## Abbreviations

CI: confidence interval; HMG-CoA: 3-hydroxy-3-methylglutaryl-coenzyme A; HR: hazard ratio; NSCOT: National Study on the Costs and Outcomes of Trauma; OR: odds ratio; RR: relative risk.

## Competing interests

The authors declare that they have no competing interests.

## Authors' contributions

JOJ conceived the study, conducted the electronic searches, reviewed the results, assessed the quality of included studies, and contributed to the writing of the manuscript. JML contributed to the writing of the manuscript. DRT and MJM helped to review the results of the electronic searches, assessed the quality of included studies, and contributed to the writing of the manuscript. DFM contributed to the writing of the manuscript. FG assisted with the conception and design of the study and contributed to the writing of the manuscript.
